# Testing persuasive messaging to encourage COVID-19 risk reduction

**DOI:** 10.1371/journal.pone.0264782

**Published:** 2022-03-23

**Authors:** Scott E. Bokemper, Gregory A. Huber, Erin K. James, Alan S. Gerber, Saad B. Omer

**Affiliations:** 1 Institution for Social and Policy Studies, Yale University, New Haven, Connecticut, United States of America; 2 Center for the Study of American Politics, Yale University, New Haven, Connecticut, United States of America; 3 Department of Political Science, Yale University, New Haven, Connecticut, United States of America; 4 Institute for Global Health, Yale University, New Haven, Connecticut, United States of America; 5 Department of Internal Medicine, Infectious Diseases, Yale School of Medicine, New Haven, Connecticut, United States of America; 6 Yale School of Public Health, New Haven, Connecticut, United States of America; Bucharest University of Economic Studies, ROMANIA

## Abstract

What types of public health messages are effective at changing people’s beliefs and intentions to practice social distancing to slow the spread of COVID-19? We conducted two randomized experiments in summer 2020 that assigned respondents to read a public health message and then measured their beliefs and behavioral intentions across a wide variety of outcomes. Using both a convenience sample and a pre-registered replication with a nationally representative sample of Americans, we find that a message that reframes not social distancing as recklessness rather than bravery and a message that highlights the need for everyone to take action to protect one another are the most effective at increasing beliefs and intentions related to social distancing. These results provide an evidentiary basis for building effective public health campaigns to increase social distancing during flu pandemics.

Governments and public health officials have emphasized the importance of social (physical) distancing and other related measures in mitigating the spread of COVID-19. Given ongoing vaccine hesitancy, that vaccines are not fully effective in preventing COVID-19 infections, and the lack of vaccine access in certain parts of the world, the need for interventions that cause individuals to take actions that reduces the risk of infection remain essential. In practice, many messaging and communication strategies have been observed. However, despite these widespread and varied efforts, we lack a robust evidentiary basis for understanding the messages that are effective at increasing individuals’ willingness to embrace actions that reduce the spread of COVID-19.

We conducted two experiments to examine how different public health messages affect people’s beliefs about the efficacy of social distancing, their intentions to practice social distancing, and their attitudes about enforcing social norms, such as persuading others to practice social distancing and negatively judging those who do not. Experiment 1 was exploratory in nature and tested a large number of messages that combined elements from different conceptual frameworks discussed below in an effort to find messages that increased respondents’ intentions to practice social distancing and willingness to encourage others to do so.

In Experiment 2, we take the two most successful messages from Experiment 1 and conduct a preregistered trial using a nationally-representative sample of American adults against both a Baseline Informational control similar to that used in Experiment 1 and a placebo-treated control group that is not exposed to any information about COVID-19 risk reduction. In our second study, in light of ongoing discussions about other practices to reduce the spread of COVID-19, we also examined mask wearing, willingness to self-isolate if exposed to COVID-19, and cooperation with government contact tracing. In both studies, we examine the possibility that certain messages are more effective among specific segments of the population.

This paper offers three important contributions. First, we conduct a large-scale multi-message study of different messages designed to encourage COVID-19 risk reduction actions with multiple outcomes followed by a replication study of the most promising messages. Testing a large number of messages means we can directly assess the relative effectiveness of different messages, decompose compound messages into their component parts to understand which elements of those messages make them effective, and address concerns that prior studies testing individual messages and finding them effective are driven by false positives. Our repeat testing of promising messages also allows us to understand whether messages that are initially effective remain effective, helping to further rule out sampling variability and understand the durability of apparently effective messages in light of changing public rhetoric about COVID-19 [1]. Finally, our focus on multiple outcomes means that we can understand both whether messages are effective only for the targeted individual’s own risk reduction behavior or also affect their likelihood of encouraging others to undertake these protective behaviors.

Second, we test a large number of different messages, drawn from three broad and theoretically relevant categories. First, we test messages that differ in whether they frame social distancing as a self- or other-regarding action and whether they highlight reciprocity in producing desirable outcomes. While several other papers have considered other-regarding messages, we also explicitly test whether it is easier to promote other-regarding behavior when highlighting reciprocity—that is how the other-regarding behavior of other individuals is also helping to protect the person targeted for persuasion. Second, we test a set of messages we characterize as “values consistent.” These are messages that try to frame social distancing in terms of values individuals likely hold, so that individuals who might otherwise be resistant to the behavior undertake it. We also test messages observed in public health and political rhetoric at the time these studies were fielded. In all cases, we test these messages relative to both a pure control that does not provide any COVID-19 relevant content and to a baseline public health message that provides a simple informational basis for social distancing as well as an injunctive appeal for doing so. This latter comparison provides further leverage in isolating the effects of any novel persuasive rhetoric.

Finally, these messaging studies provide an important window into the efficacy and limitations of efforts to promote COVID-19 risk reduction in the early stages of the pandemic in the United States and as it later evolved. Existing work on public health messaging has demonstrated behavioral change in response to specific messages about tobacco use, consumption of sugary beverages, high risk sexual behavior, and vaccination uptake [2–6]. Messages used in past work often target one or a very small number of behaviors at a time. However, successful public health strategies that address the COVID-19 pandemic require large numbers of people to change a broad range of daily behaviors, such as how they interact with friends and relatives, whether they wear face coverings in public, and cooperation with government efforts to identify infectious individuals. This suggests that a more fruitful messaging strategy needs to change attitudes towards social distancing more broadly rather than targeted messaging to increase the prevalence of a specific action. Changes in attitudes could also increase the willingness of individuals to encourage others to engage in these behaviors—that is, to reinforce desired behaviors through social norms [7–9]. Importantly, unlike other health behaviors, many individuals are at a relatively low risk of serious COVID-19 complications, but their behavior is nonetheless important for reducing the risk to individuals who are more vulnerable as the disease continues to spread throughout the general population.

Before proceeding, we note that we use the term social distancing rather than physical distancing as it reflects the language at the time the experiments were fielded. As has been noted by other researcher, the term physical distancing may be more appropriate [10–14].

## Background

The emergence of COVID-19 created an urgent need for governments and public health officials around the globe to induce behavioral change among people in society writ large. While formal restrictions, like closing schools, prohibiting large gatherings, and restricting travel, can quickly produce behavioral change, slowing the spread of infectious diseases also requires voluntary action by individuals like working from home, avoiding dining inside restaurants, and refraining from socializing with friends and family. An important challenge for public health officials is persuading people to change a large number of behaviors that cause a significant disruption to daily routines.

Given the novelty of social distancing in the United States early in the pandemic and the large number of people being told to distance to keep themselves, their families, and their community safe, it was not clear ex ante what types of messaging strategies would be effective at increasing people’s willingness to dramatically change their daily lives. While considerable work on public health messaging has been produced during the pandemic, in the early stages it was important to understand whether any component of the “kitchen sink” messages observed being used could be effective at increasing people’s beliefs about the importance of social distancing and their intentions to engage in the behavior.

The large number of messages we tested were motivated by different approaches in behavioral science. Specifically, we combined appeals about 1) social norms, 2) self-interest vs. other-regarding motives, 3) individual vs. collective action, and 4) values reframing, to better understand whether attitudes toward social distancing could be changed with written persuasive messages.

### Social norms and health behaviors

Public health campaigns often invoke social norms to encourage the public to practice positive health behaviors, like wearing sunscreen [15, 16], quitting smoking [17], and using condoms [18] (see also [19]). Beliefs about social norms have been shown to be powerful motivators of health behavior (for review, see [20]). Unsurprisingly, social norms theory has been applied to understanding people’s behaviors during the COVID-19, such as the decision to wear a mask [7, 8] and whether to practice social distancing [9, 21–23]. Social norms can be classified as either *descriptive*, i.e. what most people do, or *injunctive*, i.e. people’s beliefs about what they should do or what is believed to be the morally acceptable thing to do [24].

Early in the pandemic, public health experts had to rely on appealing to injunctive norms, emphasizing what most people *should* be doing to stay safe. Prior to COVID-19 infection becoming widespread in the United States, most people were not engaging in social distancing making it difficult to credibly appeal to descriptive norms as a way to increase the prevalence of the behavior. An appropriate baseline for comparison of messaging strategies about social distancing is therefore one that includes an appeal to injunctive norms, an approach that was relatively common at the beginning of the pandemic. Our baseline message therefore explains that public health officials believe individuals ought to socially distance to end the COVID-19 pandemic and details the specific health behaviors that people should undertake.

However, as social distancing became more widespread in the early months of the pandemic, public health messaging could also emphasize descriptive norms in conjunction with injunctive norms. For both social distancing and mask wearing, people report being more likely to engage in a public health promoting behavior when they report that others around them are doing so as well [7, 9]. Descriptive social norms may also play a causal role in the decision to wear a mask. In a vignette-based experiment, respondents in the United States and Italy were more likely to report that they would wear a mask or ask someone to wear theirs properly when other people were described as wearing masks compared to when they were not [8]. This positive effect has also been observed when accounting for local ordinances and has been shown to be stronger when people also endorse the injunctive norm that social distancing is the morally correct behavior [25]. Thus, the combination of an injunctive norm with a descriptive norm may be especially likely to increase people’s willingness to engage in social distancing.

### Self-interest vs. prosocial concern for social distancing

Descriptive social norms provide information about the prevalence of a behavior in a group of people, but this does not provide information as to why others are engaging in the behavior per se. That is, people may be practicing social distancing to protect themselves from contracting COVID-19, or they may also be practicing social distancing to protect others. It could also be that people are motivated by some combination of both motives. Past research has observed that both a concern for one’s own health and a concern for the health of others are motivations for social distancing behavior. In a survey of adults in North America and Europe, over 80% of respondents reported that they practice social distancing to protect themselves and to protect others [26]. Both motivations were also shown to be predictive of social distancing behavior in a computer-based scenario experiment in which participants reported their social distancing behavior in common daily situations, like meeting a friend or going to a grocery store [27]. Regarding concern for one’s own health, people who believe that they are more vulnerable to the disease are more likely to report higher levels of social distancing behavior [28–30]. Survey research has also examined the correlation between individual differences in personality and values has found that people who are more concerned about the well-being of others are more likely to engage in social distancing [31–34] and that this concern for others may be more predictive of behavior than concern for oneself [35]. Further, people who were less willing to place risk on others in an incentivized experiment were more likely to report engaging in social distancing than those who placed another individual at greater risk [36].

While both self-interested and prosocial motives are present in people’s decisions to engage in social distancing, research on persuasion and public health messaging has produced mixed results for the effectiveness of appealing to either motive on behavioral intentions related to social distancing. Posters highlighting an “identifiable victim” or the spread of the disease to many others have been shown to decrease the willingness to engage in behaviors that were thought to spread COVID-19, like meeting with a friend or relative in their house [37]. Other work has found that inducing empathy for someone who is particularly vulnerable to COVID-19 can increase social distancing intentions [38]. Jordan, Rand, and Yoeli [39] observed that a prosocial framing of social distancing on a flier, i.e. avoid spreading coronavirus, was more effective than a self-interested frame, i.e. avoid getting coronavirus, in March 2020, although the prosocial frame was no more effective than the self-interested frame in a related experiment fielded a month later. Prosocial and empathy-inducing messages delivered as text have also been shown to be no more effective than the informational control to which they were added [40]. Thus, it is not clear whether persuasive messaging that appeals to protecting oneself or protecting others consistently produces the intended behavioral change beyond simply providing people with information.

### Individual action vs. collective action

Descriptive social norms also do not convey how individual actions produce a benefit. Fundamentally, an outcome can be produced by individual or collective action, and the nature of a cooperative production function can differ substantially. In the case of individual production, public health campaigns could emphasize that each individual’s action produces a benefit. This approach aligns with past work on how beliefs about self-efficacy, an individual’s belief that they have the ability to perform an action to bring about a specific outcome, are an important determinant of whether an individual will perform a positive health behavior [41, 42]. Beliefs about self-efficacy have been associated with intentions to practice social distancing in response to COVID-19 [43, 44] and a hypothetical flu pandemic [45]. Thus, public health messaging may emphasize the importance of individual action as a means of protecting oneself and protecting others against COVID-19.

Alternatively, public health appeals could instead emphasize that the overall success of social distancing depends on collective action. Social distancing can be thought of as a collective action problem in which people have to work together to produce a group benefit. These types of cooperation dilemmas are widespread in human society and they vary in how the successful provision of a collective benefit is achieved [46]. One important feature of arguments that combine cooperative production with descriptive norms is that they invoke notions of reciprocity, the idea that one’s (costly) actions are being reciprocated by others in society, a factor that is shown to increase a willingness to undertake costly action [47–49].

The mapping between cooperative actions and outcomes may also vary. For one, social distancing to reduce the spread of COVID-19 could be thought of as a linear public good in which each individual’s social distancing provides an additional benefit to others. In this view, even if many people do not practice social distancing, those who do will still provide some benefit, although the fact each person’s actions matter may also encourage free-riding. Alternatively, social distancing could be thought of as a threshold public good in which the benefits are not realized until a critical mass of individuals engage in the behavior [50]. In this case, the possibility of not reaching a critical threshold may counteract the tendency to free-ride, although if the number of individuals falls short of the threshold, the benefit of social distancing is not produced and so one’s willingness to act may depend on believing enough other people are doing so.

### Values reframing

One limitation of norm based approaches for policymakers and public health officials is that some people believe that COVID-19 does not pose a threat [27, 51] or that social distancing violates another value they care about, such as displaying bravery rather than living in fear, an argument that appeared in the rhetoric of then President Donald Trump [52, 53]. Rather than attempting to convince people with these beliefs about the threat posed by COVID-19, it may instead be effective when trying to persuade them to social distance to instead frame the action of social distancing as aligning with a value that they already hold [54]. For instance, bravery and risk-taking are generally viewed as attractive traits across a variety of cultures [55–57]. And indeed, many individuals, like medical professionals and emergency responders, demonstrated these desirable traits during the COVID-19 pandemic. Is reframing the act of social distancing as demonstrating an individual’s strength and bravery an effective strategy? A values-based approach has been shown to be effective at increasing attitudes toward masking among American conservatives when messaging appealed to loyalty moral values [58]. More broadly, other work has considered how metaphors can be useful ways to frame responses to the pandemic in ways that people can easily relate to [59].

## The present experiments

We present results from two experiments that combined elements of the theoretical approaches describe above to assess the efficacy of persuasive messages to increase people’s willingness to practice social distancing.

In Experiment 1, we tested the efficacy of a large number of messages against a Baseline Informational control message that defined social distancing and stated that public health experts believe it would reduce the spread of COVID-19. We note that this message also invoked an injunctive norm because it states public health experts believe people ought to be social distancing. This was a more conservative approach than testing against an untreated control group, which we chose because we were searching for promising messages that could outperform the baseline content most similar to extant public health outreach and to which they were added in the experimental context. Our focus in Experiment 1 is to examine whether any message outperforms that Baseline Informational content to which it was added.

In Experiment 2, we re-tested the two most promising messages from Experiment 1 on a nationally-representative sample of Americans against the Baseline Informational control and a separate placebo control message.

## Experiment 1

### Methods

Participants were randomly assigned to read a Baseline Informational message or to one of ten intervention messages. Due to the number of comparisons that utilize the baseline message, we assigned participants to this message with a 3/13 chance, while the remaining ten intervention messages each had a 1/13 chance of assignment. The survey was administered using Qualtrics survey software. Both experiments presented here were fielded under an exemption granted by the Yale IRB and written consent was obtained before participants could begin the study.

### Study sample

We used a self-service online platform provided by the survey firm Lucid to recruit a sample of American adults (*n* = 3,184). Lucid provides a diverse sample of respondents that more closely matches demographic characteristics of nationally representative samples than other survey platforms like Amazon Mechanical Turk [60]. Approximately 81% of respondents assigned to an intervention completed the survey. Attrition was lower among those assigned to most of the intervention messages apart from the Baseline Informational message, by up to 8 percentage points. We did not find that pre-treatment covariates that explain outcomes differentially predicted attrition. The final analyzed sample was 2,568 respondents.

### Treatments

Participants were randomly assigned to read a Baseline Informational message that defined social distancing and stated that public health experts believe it would reduce the spread of COVID-19 or to one of ten intervention messages grouped into three categories. Each intervention message was added to the Baseline Informational message that included an injunctive norm statement. Table 1 shows the full text of the treatment messages and displays which parts of each tap into various theoretical constructs.

**Table 1:** Experiment 1 treatment messages and theoretical underpinnings.

Short Name	Treatment Text	Theoretical Underpinning
Baseline Informational Control	To end the COVID-19 pandemic, public health officials believe we should practice social distancing.	Injunctive statement
	Social distancing means that you should:	
	• Avoid gatherings of any size outside your household, such as a friend’s house, parks, restaurants, shops, or any other place.	Information about how to practice social distancing
	• Work from home when possible.	
	• Stay at least 6 feet away from others If you need to go out in public, for example to shop for food or medicine. Also consider covering your mouth and nose with a cloth face covering when around others, including when you have to go out in public.	
	• Avoid using any kind of public transportation, ridesharing, or taxis, if possible.	
Protect Self, Individual Action	Stopping COVID-19 is important because it reduces the risk that you could get sick and die.	Self-interest
	COVID-19 kills people of all ages. Even for those who are young and healthy, there is a risk of death or long-term disability.	Increases the salience of risk
	Many other people are already social distancing.	Descriptive social norm
	Remember, when you practice social distancing you reduce the risk that you get sick. Your choices affect you.	Individual action + self-interest
Protect Self, Linear Cooperation	Stopping COVID-19 is important because it reduces the risk that you could get sick and die.	Self-interest
	COVID-19 kills people of all ages. Even for those who are young and healthy, there is a risk of death or long-term disability.	Increases the salience of risk
	Many other people are already social distancing.	Descriptive social norm
	Remember, every person who practices social distancing reduces the risk that you get sick. While you can’t do it alone, we can all protect ourselves by working together.	Linear cooperation + self-interest
Protect Self, Threshold Cooperation	Stopping COVID-19 is important because it reduces the risk that you could get sick and die.	Self-interest
	COVID-19 kills people of all ages. Even for those who are young and healthy, there is a risk of death or long-term disability.	Increases the salience of risk
	Many other people are already social distancing.	Descriptive social norm
	Remember, if enough people practice social distancing then we can reduce the risk that you get sick. While you can’t do it alone, we can protect ourselves if enough of us work together.	Threshold cooperation + self-interest
Other-regarding, Individual Action	Stopping COVID-19 is important because it reduces the risk that members of your family and community could get sick and die.	Prosocial motives
	COVID-19 kills people of all ages. Even for those who are young and healthy, there is a risk of death or long-term disability.	Increases the salience of risk
	Many other people are already social distancing.	Descriptive social norm
	Remember, when you practice social distancing you reduce the risk that people you care about get sick. Your choices affect those around you.	Individual action + prosocial motives
Other-regarding, Linear Cooperation	Stopping COVID-19 is important because it reduces the risk that members of your family and community could get sick and die.	Prosocial motives
	COVID-19 kills people of all ages. Even for those who are young and healthy, there is a risk of death or long-term disability.	Increases the salience of risk
	Many other people are already social distancing.	Descriptive social norm
	Remember, every person who practices social distancing reduces the risk that people you care about get sick. While you can’t do it alone, we can all protect everyone by working together.	Linear cooperation + prosocial motives
Other-regarding, Threshold Cooperation	Stopping COVID-19 is important because it reduces the risk that members of your family and community could get sick and die.	Prosocial motives
	COVID-19 kills people of all ages. Even for those who are young and healthy, there is a risk of death or long-term disability.	Increases the salience of risk
	Many other people are already social distancing.	Descriptive social norm
	Remember, if enough people practice social distancing then we can reduce the risk that people you care about get sick. While you can’t do it alone, we can protect our loved ones if enough of us work together.	Threshold cooperation + prosocial motives
Reframing Bravery	Soldiers, fire fighters, EMTs, and doctors are putting their lives on the line to serve others during the COVID-19 pandemic. That’s bravery.	Examples of bravery
	But people who don’t practice social distancing because they don’t think they will get sick or aren’t worried aren’t brave, they are reckless. By not social distancing, you are risking the health of your family, friends, and community.	Reframing not social distancing as not bravery, but recklessness
	There is nothing attractive and independent-minded about ignoring public health guidance to practice social distancing. Not social distancing means you risk the health of others. To show strength practice social distancing so you don’t get sick and take resources from other people who need them more, or risk spreading the disease to those who are at risk. Social distancing may be inconvenient, but it works.	Not social distancing is not attractive and puts others at risk
Reframing Bravery + Pollution	Soldiers, fire fighters, EMTs, and doctors are putting their lives on the line to serve others during the COVID-19 pandemic. That’s bravery.	Examples of bravery
	But people who don’t practice social distancing because they don’t think they will get sick or aren’t worried aren’t brave, they are reckless. By not social distancing, you are risking the health of your family, friends, and community by polluting the community with the risk of infection.	Reframing not social distancing as not bravery, but recklessness and polluting with risk of infection
	There is nothing attractive and independent-minded about ignoring public health guidance to practice social distancing. Not social distancing means you risk the health of others. To show strength practice social distancing so you don’t get sick and take resources from other people who need them more, or risk spreading the disease to those who are at risk. Social distancing may be inconvenient, but it works.	Not social distancing is not attractive and puts others at risk
Return to Normal	Other-regarding, Linear Cooperation + Social distancing now means we can more quickly return to our normal way of life.	Contemporary appeal
New Normal	Other-regarding, Linear Cooperation + Social distancing now means we are adapting to the "new normal" necessary because of COVID-19.	Contemporary appeal

This table shows the elements of each message that correspond to a theoretical construct that was discussed above.

The first category of messages varied the beneficiary of social distancing behaviors and whether individual or collective action was needed to produce these benefits. In all of these messages, descriptive social norms were invoked by describing others as already social distancing (“Many other people are already social distancing.”). The beneficiary of social distancing was either the individual (“you could get sick and die”) or others (“members of your family and community could get sick and die”). We combined manipulation of the beneficiary with what was necessary to produce this benefit. Specifically, social distancing was framed as providing a benefit if an individual practiced it (individual action, “when you practice social distancing you reduce the risk”), if *enough* other people practiced it (threshold collective action, “if enough people practice social distancing then we can reduce the risk”), or for each additional person who practiced (linear collective action, “every person who practices social distancing reduces the risk”). As we note above, the latter two frames about collective production also emphasized norms of reciprocity in that they linked others’ behaviors to outcomes relevant for the respondent. Crossing these two dimensions of manipulation produced the six total intervention messages in this category.

The second category of messages were efforts at value reframing and stated that people who believe they are being brave by continuing with their daily routines despite the threat of the virus are actually being reckless. Theses message start with an example of people who are being brave during the pandemic, e.g. firefighters, and then takes a seemingly desirable action as incompatible with a value and reframes it instead as selfish and unattractive (“people who don’t practice social distancing… aren’t brave, they are reckless”). The message also emphasizes that by not social distancing, people are placing others at risk, i.e. the opposite of true bravery. This reframing was either presented alone (Reframing Bravery) or with language about how people who spread COVID-19 pollute the environment around them (Reframing Bravery + Pollution).

The final category of messages invoked the idea that practicing social distancing would facilitate returning to “normal” life before the COVID-19 pandemic (“Social distancing now means we can more quickly return to our normal way of life”) or that doing so involved adapting to an unavoidable “new normal” (“we are adapting to the ‘new normal’ necessary because of COVID-19”). These two messages were designed to mirror rhetoric being used by political leaders and in the media and were added to the Other-regarding, Linear Cooperation message.

### Outcomes

We form four mean scales as outcome measures, with all scales ranging from 0 to 1 with 1 indicating behaviors or beliefs associated with reducing the spread of COVID-19. The four scales were: 1) a BELIEFS/norms scale that assesses agreement with beliefs about social distancing being important for your health and others people’s health and whether an individual would feel guilty for not practicing social distancing, 2) a social distancing (DISTANCING) scale that captures people’s intended willingness to social distance, avoid attending gatherings, forego elective medical procedures, and wear a mask, 3) a FOOD behavior scale that assesses people’s willingness to avoid high-risk food related behavior like going to a restaurant, and 4) a persuade/evaluate OTHERS scale that measures whether people would persuade others to social distance, report a business for violating rules, and negatively judge non-compliers. Several items in the DISTANCING and FOOD scales had previously been shown to be affected by rhetoric focusing on selfish and prosocial motivations for social distancing [39]. All of the outcomes were coded such that higher values corresponded to attitudes and behavioral intentions consistent with greater social distancing. The internal consistency of the scales was generally good with the exception of the FOOD scale, which had a Cronbach’s Alpha of 0.65. Full text of outcome measures and scaling information appears in S1 Appendix.

### Results

**Fig 1:**
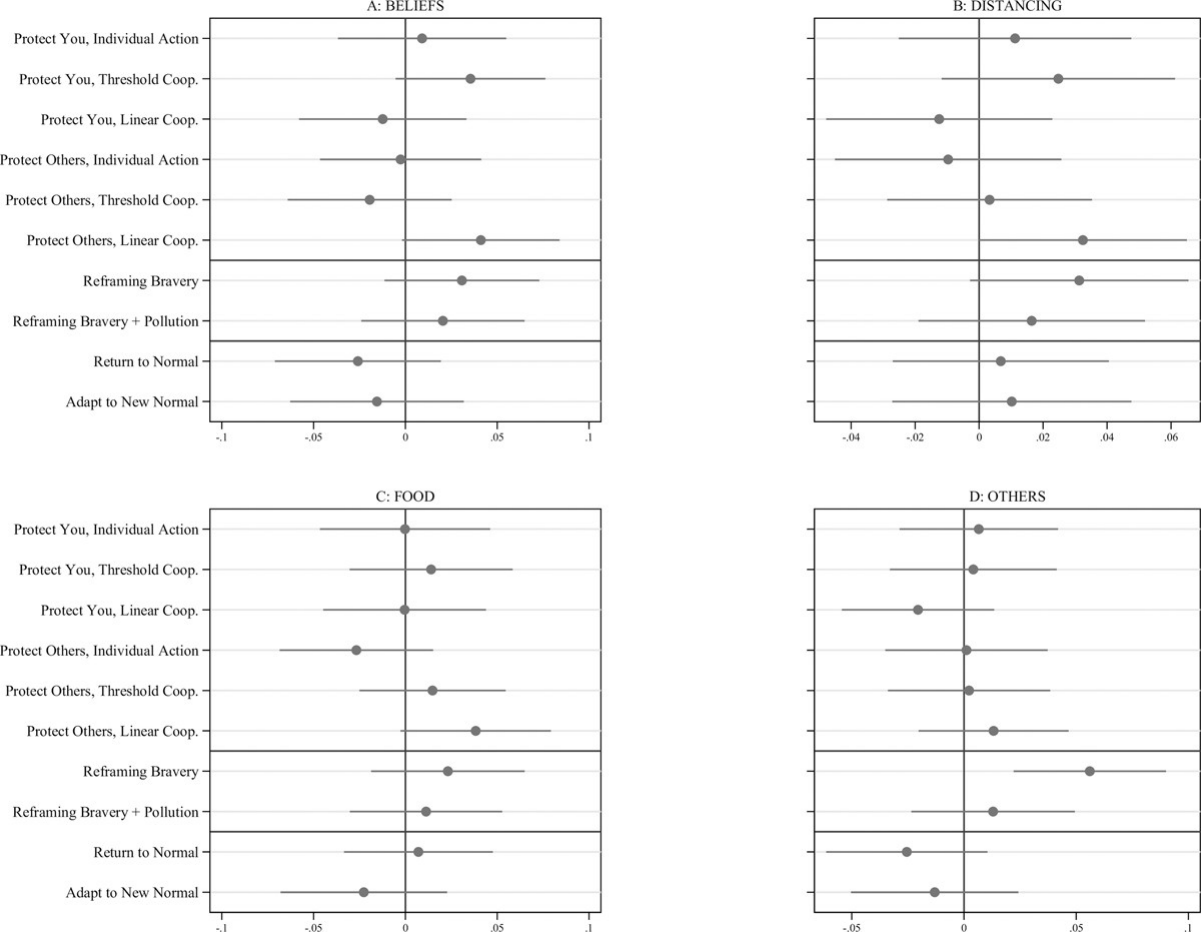
Covariate-adjusted treatment effects estimated using OLS regression with robust Huber-White standard errors. Estimates displayed with 95% confidence intervals. Each panel shows the effect of each treatment message relative to the Baseline Informational condition for a primary outcome scale. All outcomes scales were coded such that higher values indicate more positive attitudes or intentions toward social distancing.

We analyze our data using OLS regression comparing outcomes to the Baseline Informational message using indicators for each treatment and including pre-treatment demographic covariates to improve efficiency. Two messages appear particularly promising compared to the Baseline Informational message, with all treatment effect estimates plotted in Fig 1. (Underlying regression analysis and distribution of scale outcomes appears in the S2 Appendix). Among the messages that appear most effective, the Other-regarding, Linear Cooperation message also performs well relative to the Baseline Informational condition. For all four scales, the estimated effects of this message are positive. For the social distancing scale, the effect is .034 (95% CI: .002, .067) or 14.7% of a standard deviation. Respondents’ beliefs about the importance of social distancing also increase with an estimated effect of .040 (95% CI: -.002, .084) on the BELIEFS scale. The effect on the FOOD scale is .038 (95% CI: -.003, .079). The latter two effects are not statistically significant at the conventional 5% level, but do provide evidence that the Other-regarding, Linear Cooperation message broadly moved beliefs relevant to practicing social distancing.

The Reframing Bravery message increases all four scale outcomes. The estimated effect on the OTHERS scale is .058 (95% CI: .023, .092), indicating that respondents who read the Reframing Bravery message report more willingness to enforce norms to promote social distancing. We also observe suggestive evidence that this message affects both the BELIEFS scale and the own social distancing scale. For the BELIEFS scale the estimate is .037 (95% CI: -.005, .079) or about 12.8% of a standard deviation, while the effect for the DISTANCING scale is .030 (95% CI: -.004, .064) or about 13% of a standard deviation. The estimated effect for the FOOD scale is positive, but imprecise.

It is also interesting that two messages appear, on average, less effective than the Baseline Informational content and the Other-regarding, Linear Cooperation message to which they are added. While no coefficient estimates are individually statistically significant, both the Return to Normal and New Normal messages are generally less effective than the content to which they were added across our primary outcome measures.

We also conduct a number of exploratory analyses for heterogeneous treatment effects by age, gender, partisanship, and geographic location and do not uncover large differences in average treatment effectiveness across these groups (S3 Appendix). Due to the rhetoric among the public and political elites surrounding the degree to which measures to address the spread of COVID-19 infringe upon people’s liberties, we elicited people’s adoption of a liberty moral foundation that captures their belief about the role of government in society [61]. We found evidence that intervention effectiveness varies by endorsement of liberty values. Compared to respondents below the mean in their adoption of liberty values, respondents who are above the mean in their adoption of liberty are more responsive to the Reframing Bravery message than to the Baseline Informational condition on the BELIEFS scale (p = .05) and OTHERS scale (p < .01), with weaker evidence for the DISTANCING scale (p = .14). The effects of the Reframing Bravery message are uniformly statistically insignificant for those low in liberty.

### Discussion

The two most promising messages were the Other-regarding, Linear Cooperation message and the Reframing Bravery message. Both were the highest performing messages on at least two of the four outcome scales when compared to the baseline content to which they were added. Given this, these messages were the ones that were selected to be re-tested on a nationally representative sample of Americans to discern whether they are more effective than the Baseline Informational content to which they were added. Additionally, we believe there was value in retesting the most effective messages at a later point in the time in the pandemic when attitudes about social distancing may have become more crystallized, perhaps making people harder to persuade.

## Experiment 2

### Methods

Experiment 2 retested the two most successful interventions in Experiment 1 (Reframing Bravery, and Other-regarding Linear Cooperation and the Baseline Informational compared to an untreated Control message about an unrelated topic (bird feeding)). Experiment 2 was a pre-registered trial fielded between mid-July and early August 2020, a time when the COVID-19 outbreak in the United States had become far more widespread than during Experiment 1 [62]. We allocated respondents with equal probability to each intervention and written consent was obtained prior to participation.

### Study sample

We used the survey firm YouGov to recruit a nationally-representative sample of American adults. Respondents completed the study on their personal electronic devices. Power calculations indicated greater than 80% power to detect treatment effects 75% as large as in Experiment 1 with an N of 3,000 assuming scale distributions were the same as observed in Experiment 1. The study was fielded twice because of an implementation error in programming by the vendor for survey content that followed the items analyzed here for the first fielding (the error was for items for an unrelated project that was not about COVID-19, and which followed all of the items analyzed here). Consequently, the vendor re-fielded the entire survey resulting in a sample that was approximately twice as large as the sample described in our pre-registration document (*n* = 3,000 pre-registered, *n* = 6,079 in final analysis dataset). YouGov does not provide data for respondents who decline to participate or drop out during the study.

### Treatments

The Baseline Informational treatment message was slightly modified from Experiment 1 to reflect changing guidance during the pandemic. It read:

To end the COVID-19 pandemic, public health officials believe we should practice social distancing. Social distancing means that you should:

- Work from home when possible
- Wear a mask that covers your nose and mouth when outside of your home around other people
- Stay at least 6 feet away from others if you need to go out in public, for example to shop for food or medicine
- Avoid large gatherings, especially indoors
- Stay home except to seek medical care if you are sick or have recently had close contact (closer than 6 feet for at least 15 minutes) with a person with COVID-19
- Avoid pooled rides or rides where multiple passengers are picked up who are not in the same household

The additional content added to this baseline for the Other-regarding, Linear Cooperation and the Reframing Bravery messages was unchanged from how they appear in Table 1.

### Outcomes

We made incremental changes to the four scales (BELIEFS, DISTANCING, FOOD, and OTHERS) used in Experiment 1 to reflect changing policies and circumstances. Given that contemporary discourse around social distancing had changed, we included new items that reflected what people were likely thinking about in their daily lives. We added items to the DISTANCING scale about attendance at religious services, participation in political events, self-isolation following COVID-19 exposure, and alerting public health authorities if diagnosed with COVID-19. For the OTHERS scale we added an item about cooperating in contact tracing. In the months between our studies, the behaviors we added to the scales had become salient in public discourse about COVID-19 risk reduction. We also included a new MASK scale composed of items about wearing a face covering in six circumstances, as well as relative willingness to shop at a store that requires rather than prohibits face masks. These additional items (and perhaps the passage of time) increased the reliability of the four scales that were used in Experiment 1 with the FOOD scale having the lowest reliability (Cronbach’s alpha of 0.78). The modified outcome text and scale reliability appears in S4 Appendix.

At the time this experiment was fielded, messaging outside of the experimental context about the importance of items in our DISTANCING scale had become far more widespread, although mask wearing remained a contested policy tool. It was therefore unclear whether messaging would be similarly effective in this new context.

### Results

**Fig 2:**
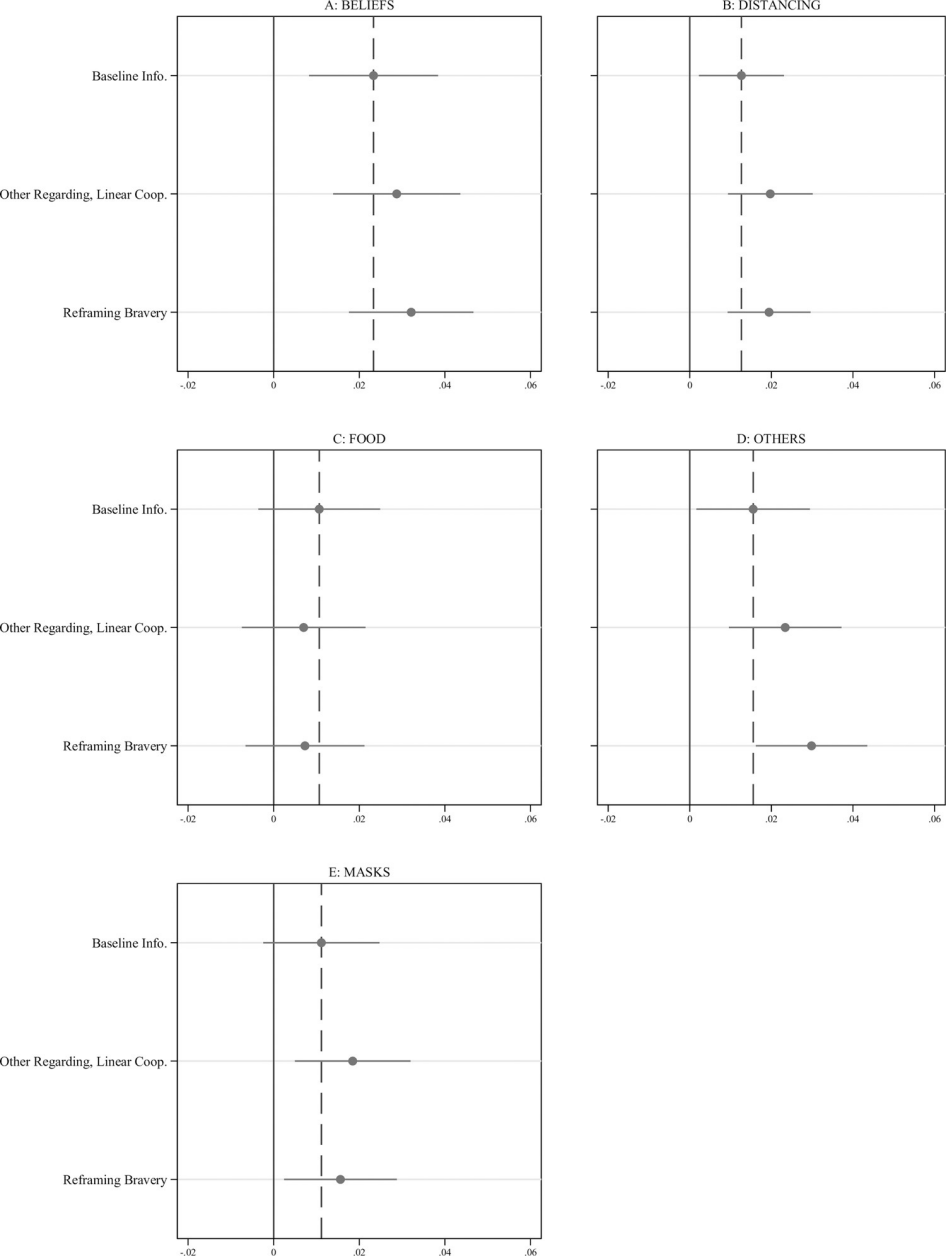
Experiment 2 results. Compared to the placebo control, the Baseline Informational message, the Reframing Bravery message, and the Other-regarding, Linear Cooperation increase beliefs and reported behavioral intentions to practice social distancing. These are OLS regression coefficient estimates for each primary outcome by treatment compared to the placebo control with 90% confidence intervals. The dashed vertical line represents the effect of the Baseline Informational Message on an outcome. All outcomes scales were coded such that higher values indicate more positive attitudes or intentions toward social distancing.

We find baseline increases in scores on the BELIEFS and DISTANCING scales over time (i.e., averages for these outcomes in the bird feeding Control message in Experiment 2 are greater than the averages in the Baseline Informational condition in Experiment 1). Fig 2 plots main effects of message efficacy compared to the Control message for all outcomes (underlying regression analysis and distribution of scale outcomes appears in S5 Appendix). The Baseline Informational message is associated with increased BELIEFS and DISTANCING scores (p < .05, one-sided, in both cases) relative to the bird feeding message. The Reframing Bravery and Other-regarding, Linear Cooperation messages appear to be more effective, however. Each is associated with a statistically significant increase in four outcomes: the BELIEFS, DISTANCING, OTHERS, and MASKS scales, with p-values < .05, one-sided, in all cases. The magnitudes of these effects are approximately 0.1 standard deviation for each measure. None of the messages have large or statistically precise effects on the FOOD scale.

There is less clear evidence that these messages are incrementally more effective that the Baseline Informational content to which they are added. For the BELIEFS, DISTANCING, OTHERS, and MASKS scales, both the Reframing Bravery and Other-regarding, Linear Cooperation messages are associated with effects that are always larger than the Baseline Informational message, with the magnitudes of these differences ranging from 22% to 88% and averaging 50%. Because effect sizes are still modest, however, these differences are not generally statistically distinguishable at p < .05, two-sided, with the notable exception of the Reframing Bravery message which has an effect 88% larger than the Baseline Informational message on the OTHERS scale.

Differences in effects for those who endorse liberty values partially confirm Study 1 (See S6 Appendix). Compared to the Control message, the Reframing Bravery message is more effective among those who endorse liberty for encouraging social distancing—it increases DISTANCING measure by .027 units (90% CI: .009, .043), an effect that is 70% larger than the effect for those who do not endorse liberty values. This difference is not significant, however, and the estimates for the other outcomes are inconsistently signed. If we instead focus on the relative effectiveness of the Reframing Bravery message compared to the Baseline Informational message, a test that accounts for the fact that those who endorse liberty values may respond differently to the baseline content, we uncover more evidence that those who endorse liberty values respond more to the Reframing Bravery treatment. In particular, for those who endorse liberty values, the Reframing Bravery message is between 20% and 125% more effective than the Baseline message for the five primary outcomes. The largest difference is for the DISTANCING scale outcome, where the difference is .014 (90% CI: -.004, .033).

**Fig 3:**
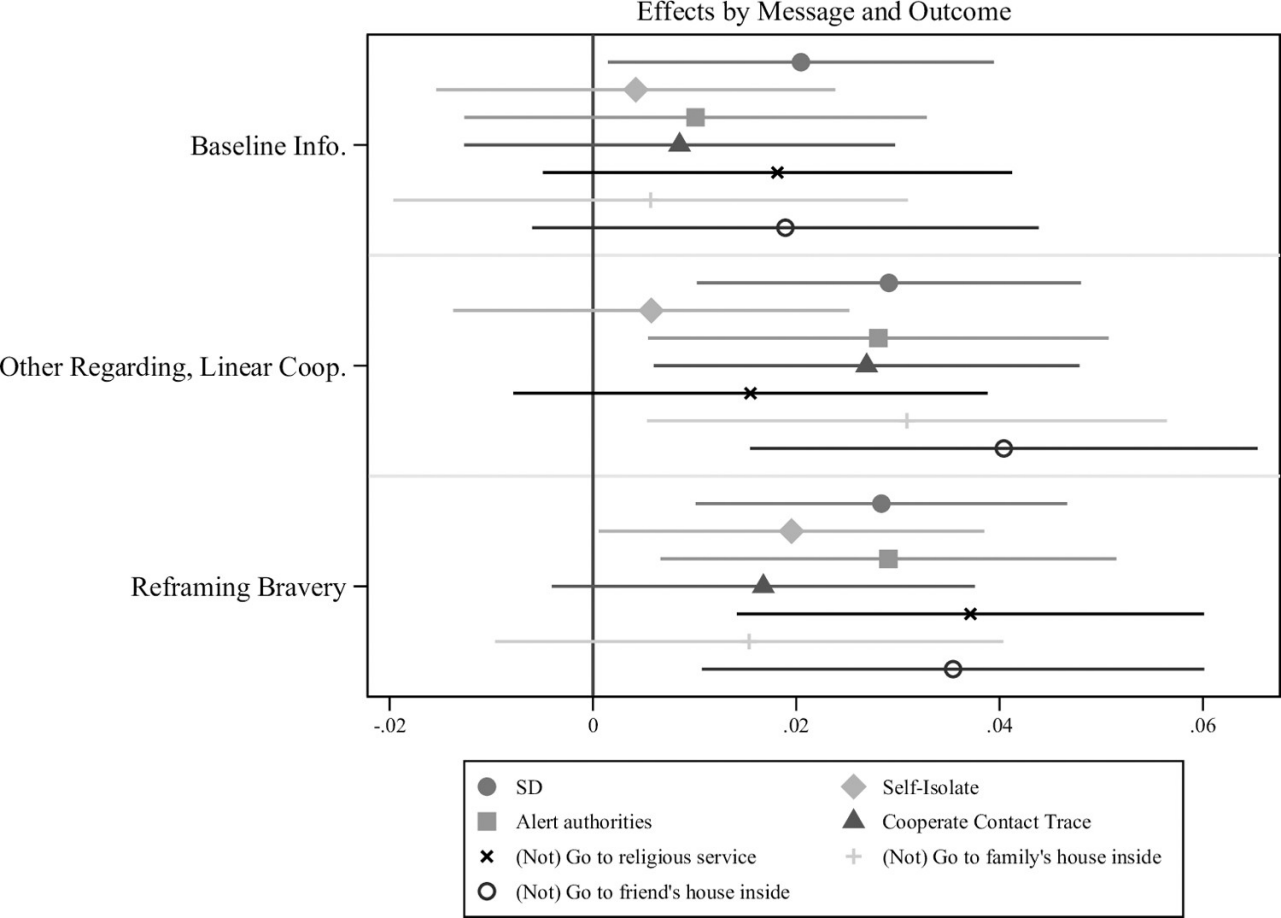
Experiment 2 individual social distancing items. The Reframing Bravery and Other-regarding, Linear Cooperation message increase respondents reported intentions to *not* engage in key behaviors to reduce the spread of COVID-19 and to cooperate with government officials, even compared to the Baseline Informational message. This figure shows OLS regression coefficient estimates compared to the Control message with 95% confidence intervals. All outcomes scales were coded such that higher values indicate more positive attitudes or intentions toward social distancing.

In addition to our scale outcomes, we also examine results for several individual items of particular interest, including the three measures of compliance with government policies to reduce the spread of COVID-19 discussed above: Self-isolation for those exposed, alerting authorities if testing positive, and cooperation with authorities in contact tracing. These items are included in the DISTANCING behavior index, but are also individually of interest because they are areas where governments have reported difficulty obtaining compliance. Fig 3 show that the Reframing Bravery message is associated with a statistically significant increase in self-isolation and willingness to alert authorities, effects that are larger than and statistically distinguishable from the effects of the Baseline Informational message. (Underlying regression results appear in S5 Appendix) Similarly, the Other-regarding, Linear Cooperation message is associated with a statistically significant increase in self-isolation and willingness to cooperate in contact tracing, effects that are larger than and statistically distinguishable from the effects of the Baseline Informational message.

Second, we also examine effects for three isolated behaviors, attendance at religious gatherings and inside visits to a friend and family member’s house. Religious gatherings emerged as sources of conflict over prohibitions on group meetings (*18*), while private indoor meetings are thought to be vehicles by which asymptomatic individuals expose those who are at more serious risk for infection. Once again, these items are individually in the DISTANCING behavior index. Results appear in Fig 3. The Reframing Bravery Message is associated with statistically significant increases in all three outcomes, while the Other-regarding, Linear Cooperation message is associated with changes in both the family and friend small gathering outcomes. The Reframing Bravery effect for attendance at religious services is statistically distinguishable from the effect of the Baseline Informational message (p < .05). The Other-regarding, Linear Cooperation effect for each type of private gatherings is also statistically larger than the effect of the Baseline Informational message (p < .03 and .05, respectively).

### Discussion

In Experiment 2 we find that the Baseline Informational message, the Other-regarding Linear Cooperation message, and the Reframing Bravery message outperform the placebo control message on the primary outcome scales, with the exception of the FOOD scale. Moreover, this experiment replicates the finding from Experiment 1 that respondents who are high in liberty values are more responsiveness to the Reframing Bravery message.

## General discussion

The results presented here show that public health messaging can increase behavioral intentions and beliefs about social distancing that helps reduce the spread of COVID-19. Specifically, we observed that an Other-regarding, Linear Cooperation message that 1) focused people on protecting others, 2) increased the salience of risk presented by COVID-19, 3) emphasized that other people were social distancing, and 4) stated that every person who practices social distancing protects others was effective at increasing attitudes and behavioral intentions related to social distancing. We also found that a Reframing Bravery message that 1) gave examples of bravery, 2) reframe not social distancing as not being brave, but being reckless, and 3) emphasized that not social distancing is not attractive and places others at risk was effective. Importantly, these messages are effective in both an initial study fielded in May 2020 and in a replication study fielded in August 2020, and this efficacy is in comparison to a Baseline Informational message communicating the factual basis for social distancing behavior and instructing others to do so. We observe these effects for measures of a respondent’s own intended social distancing activities as well as for how individuals are likely to behave toward others who do not social distance.

It is also worth noting that a simple Baseline Informational message that invoked an injunctive norm that people should be social distancing and explained what social distancing was outperformed a placebo-control condition in Experiment 2. This suggests that relatively early in the pandemic simply providing people with information and emphasizing that doing these things is the correct behavior may be enough to increase attitudes toward social distancing and behavioral intentions to do so.

Moral foundations theory, [61, 63] which postulates that humans have several underlying common values that are differentially emphasized by various individuals, has been used to explain health behaviors such as vaccination [64]. Increasingly, opposition to public health measures is grounded in the language of personal freedoms [64] and, indeed, concerns about government infringement on personal freedoms have arisen during the COVID-19 pandemic [65, 66]. We find that emphasis on liberty value modifies the impact of the Reframing Bravery intervention indicating that such messages are particularly powerful for those for whom personal freedoms are important.

A potential avenue future research could explore how messaging strategies interact with people’s motivation for social distancing. Past research has found that many people engage in social distancing to protect themselves and to protect others [26]. However, other work has observed that people who endorsed conspiracy theories were more concerned about themselves and were also less likely to report intentions to practice social distancing [67]. Given heterogeneity in people’s motivations to protect themselves or to protect others, some messaging strategies, like the Other-regarding, Linear Cooperation message, may have different effects depending on whether it aligns with the motivation that a given individual holds. More broadly, future work should consider how people’s concern for themselves and concern for others interact with how receptive they are to specific public health campaigns.

This work has several limitations that should be considered alongside the results. First, while we observe robust attitudinal change in response to persuasive messaging, we do not observe actual behavioral change. Given the relatively small effect sizes, approximately 0.1 standard deviation increases on the primary outcomes in Experiment 2, these treatment messages as written communication may be insufficient to push people to change their behavior. Second, we utilized compound treatments that invoked many different constructs that are thought to produce attitude and behavioral change. Future work should focus on disentangling whether specific elements of the messages are particularly effective at promoting social distancing. Third, policymakers and public health experts had repeatedly emphasized the importance of social distancing and survey respondents may have over-reported their intentions to social distance due to social desirability concerns, though past work has found that reported behavioral intentions correlate with actual behavior [68] and people’s self-reported behavior is not affected by social desirability bias [69]. Third, as the COVID-19 pandemic has rapidly evolved and different behaviors, like masking or vaccination, have become more salient in public discourse, the messages that we find to be effective in summer 2020 may not be as effective as the pandemic has progressed. Finally, we only measured attitudes and behavioral intentions at a single point in time so we cannot make claims about the duration of the effects that we observe.

Our findings can inform both mass public health messaging initiatives (e.g. those deployed on social and electronic media) as well as interpersonal communication strategies such as healthcare provider-level communication and persuasion. While this work shows robust attitudinal changes in response to public health messaging, additional research is necessary to determine which specific elements of the treatments produced these changes.

## Supporting information

S1 Appendix Experiment 1 outcomes. (DOCX)

S2 Appendix Regression results for Fig 1 and distribution of outcomes for Experiment 1. (DOCX)

S3 Appendix Subgroup analyses for Experiment 1. (DOCX)

S4 Appendix Experiment 2 outcome measures. (DOCX)

S5 Appendix Regression results for Figs 2 and 3 and distribution of outcomes for Experiment 2. (DOCX)

S6 Appendix Liberty endorsement subgroup analysis for Experiment 2. (DOCX)
