# Supplementary material for: Testing persuasive messaging to encourage COVID-19 risk reduction
Source: PLoS One. 2022 Mar 23;17(3):e0264782. doi: 10.1371/journal.pone.0264782 (PMC8942219; doi:10.1371/journal.pone.0264782)
Supplement: S1 Appendix — (DOCX) [file pone.0264782.s001.docx]

S1 Appendix: Experiment 1 Outcomes and Coding

*Primary Outcomes*

BELIEFS about the efficacy and importance of social distancing scale, composed of the average of the following 3 items. (Cronbach’s alpha = 0.89)

To what extent do you agree with the following statements?

Scored 0 (Strongly disagree), .25 (Somewhat disagree), .5 (Neither agree nor disagree), .75 (Somewhat agree), and 1 (Strongly agree).

1. Practicing social distancing is important so that I do not get sick

2. Practicing social distancing is important so that I do not risk infecting others or pose a burden on the health system

3. I would feel guilty if I did not practice social distancing

DISTANCING scale is composed of the average of the following 6 items. (Cronbach’s alpha = 0.78)

1. If you go out in public this week to a store or somewhere else where you have to be inside with other people, how likely are you to wear a mask?

To what extent do you agree with the following statements?

Scored 0 (Strongly disagree), .25 (Somewhat disagree), .5 (Neither agree nor disagree), .75 (Somewhat agree), and 1 (Strongly agree).

2. I am likely to practice social distancing in the future.

How likely are you to do each of the following things in the next two weeks?

Scored 0 (Extremely unlikely), .25 (Somewhat unlikely), .5 (Neither likely nor unlikely), .75 (Somewhat likely), and 1 (Extremely likely). For Reverse Coded items, scale is flipped.

3. Go to a doctor or dentist for an elective procedure (Reverse Coded)

4. Go to a friend's house (Reverse Coded)

5. Go to another family member's house (Reverse Coded)

6. Go to a small party or similar social event (Reverse Coded)

FOOD behaviors scale, composed of the average of the following 3 items. (Cronbach’s alpha = 0.65)

How likely are you to do each of the following things in the next two weeks?

Scored 0 (Extremely unlikely), .25 (Somewhat unlikely), .5 (Neither likely nor unlikely), .75 (Somewhat likely), and 1 (Extremely likely). For Reverse Coded items, scale is flipped.

1. Go to a coffee shop (Reverse Coded)
2. Go out to eat (Reverse Coded)

Would you like to see a list of restaurants in your area that are open for Dine In or Take Out service?

Yes, Dine In

Yes, Take out

No

1. The last item was coded 1 if a respondent said Yes, Take Out or No and 0 if they said Yes dine in.

Evaluations and actions toward OTHERS based on their social distancing behavior scale, composed of the average of the following 6 items. (Cronbach’s alpha = 0.78)

How likely are you to do each of the following things:

Scored 0 (Extremely unlikely), .25 (Somewhat unlikely), .5 (Neither likely nor unlikely), .75 (Somewhat likely), and 1 (Extremely likely). For Reverse Coded items, scale is flipped.

1. Report a local business that is violating rules for protecting public health, like limiting crowd sizes and maintaining appropriate social distancing

2. Persuade a friend or relative to practice social distancing if they were not already doing so

Now, we would like you to think about a friend or relative who hasn’t been practicing social distancing. What would you think about this person? Are they…

Scored 0 (Not at all), .25 (Slightly), .5 (Somewhat), .75 (Mostly), and 1 (Very). For Reverse Coded items, scale is flipped.

3. Trustworthy (Reverse coded)

4. Selfish

5. Likeable (Reverse coded)

6. Competent (Reverse coded)

*Liberty Scale*

Liberty Scale, composed of the average of the following 5 items.

How much do you agree with each of the following statements?

Scored 0 (Strongly disagree), .25 (Somewhat disagree), .5 (Neither agree nor disagree), .75 (Somewhat agree), and 1 (Strongly agree). For Reverse Coded items, scale is flipped.

1. People who are successful in business have a right to enjoy their wealth as they see fit

2. Society works best when it lets individuals take responsibility for their own lives without telling them what to do

3. The government interferes far too much in our everyday lives

4. The government should do more to advance the common good, even if that means limiting the freedom and choices of individuals (Reverse Coded)

5. People should be free to decide what group norms or traditions they themselves want to follow

The liberty scale is coded by adding scores for these items together and dividing by 5. It therefore runs from 0 to 1.
