# Supplementary material for: Testing persuasive messaging to encourage COVID-19 risk reduction
Source: PLoS One. 2022 Mar 23;17(3):e0264782. doi: 10.1371/journal.pone.0264782 (PMC8942219; doi:10.1371/journal.pone.0264782)
Supplement: S2 Appendix — (DOCX) [file pone.0264782.s002.docx]

S2 Appendix: Regression Results for Figure 1 and Distribution of Outcomes for Experiment 1

|  | (1) | (2) | (3) | (4) |
| --- | --- | --- | --- | --- |
|  | BELIEFS | OWN-SD | FOOD | OTHERS |
| Reframing Bravery | 0.037 | 0.030 | 0.019 | 0.058 |
|  | [0.021]* | [0.018]* | [0.021] | [0.017]*** |
| Reframing Bravery + Pollution | 0.021 | 0.017 | 0.012 | 0.013 |
|  | [0.023] | [0.018] | [0.021] | [0.019] |
| Self Regarding, Individual Action | 0.008 | 0.012 | 0.000 | 0.005 |
|  | [0.023] | [0.019] | [0.024] | [0.018] |
| Self Regarding, Threshold Coop. | 0.033 | 0.024 | 0.013 | 0.004 |
|  | [0.021] | [0.019] | [0.023] | [0.019] |
| Self Regarding, Linear Coop. | -0.013 | -0.012 | 0.000 | -0.021 |
|  | [0.023] | [0.018] | [0.023] | [0.017] |
| Other Regarding, Individual Action | -0.003 | -0.009 | -0.026 | 0.001 |
|  | [0.022] | [0.018] | [0.021] | [0.018] |
| Other Regarding, Threshold Coop. | -0.021 | 0.004 | 0.015 | 0.002 |
|  | [0.023] | [0.016] | [0.020] | [0.019] |
| Other Regarding, Linear Coop. | 0.040 | 0.034 | 0.038 | 0.013 |
|  | [0.022]* | [0.017]** | [0.021]* | [0.017] |
| Return to Normal | -0.023 | 0.007 | 0.007 | -0.026 |
|  | [0.023] | [0.017] | [0.021] | [0.018] |
| Adapt to New Normal | -0.017 | 0.010 | -0.023 | -0.014 |
|  | [0.024] | [0.019] | [0.023] | [0.019] |
| Age in years | 0.004 | 0.003 | 0.003 | 0.001 |
|  | [0.000]*** | [0.000]*** | [0.000]*** | [0.000]*** |
| Female (1=yes) | 0.063 | 0.057 | 0.055 | 0.028 |
|  | [0.011]*** | [0.009]*** | [0.011]*** | [0.009]*** |
| Household Income | 0.003 | 0.000 | -0.001 | 0.000 |
|  | [0.001]*** | [0.001] | [0.001] | [0.001] |
| Household Income missing | -0.098 | -0.008 | 0.027 | -0.030 |
|  | [0.031]*** | [0.023] | [0.028] | [0.022] |
| Some high school (base category) | 0.000 | 0.000 | 0.000 | 0.000 |
|  | [0.000] | [0.000] | [0.000] | [0.000] |
| High school graduate | 0.027 | 0.030 | 0.011 | 0.010 |
|  | [0.034] | [0.025] | [0.028] | [0.026] |
| Post high school vocational training | 0.002 | 0.018 | -0.056 | 0.014 |
|  | [0.043] | [0.033] | [0.040] | [0.036] |
| Some college | 0.045 | 0.036 | 0.013 | 0.020 |
|  | [0.034] | [0.026] | [0.030] | [0.027] |
| Associate’s degree | 0.020 | 0.030 | 0.011 | 0.024 |
|  | [0.037] | [0.028] | [0.032] | [0.029] |
| Bachelor’s degree | 0.044 | 0.028 | 0.005 | 0.021 |
|  | [0.034] | [0.025] | [0.029] | [0.027] |
| Masters degree | 0.065 | 0.042 | -0.036 | 0.025 |
|  | [0.036]* | [0.027] | [0.032] | [0.028] |
| Professional Degree | 0.009 | 0.011 | -0.041 | 0.047 |
|  | [0.053] | [0.041] | [0.050] | [0.039] |
| Strong Democrat | 0.058 | 0.027 | 0.008 | 0.057 |
|  | [0.018]*** | [0.014]** | [0.018] | [0.014]*** |
| Democrat | 0.070 | 0.035 | 0.040 | 0.049 |
|  | [0.020]*** | [0.015]** | [0.019]** | [0.016]*** |
| Democrat Lean | 0.043 | 0.002 | 0.021 | 0.031 |
|  | [0.023]* | [0.018] | [0.022] | [0.017]* |
| Republican Lean | -0.095 | -0.061 | -0.050 | -0.026 |
|  | [0.026]*** | [0.020]*** | [0.024]** | [0.021] |
| Republican | -0.039 | -0.063 | -0.034 | -0.067 |
|  | [0.021]* | [0.017]*** | [0.020]* | [0.018]*** |
| Strong Republican | -0.071 | -0.136 | -0.163 | -0.078 |
|  | [0.020]*** | [0.015]*** | [0.019]*** | [0.016]*** |
| Northeast (base category) | 0.000 | 0.000 | 0.000 | 0.000 |
|  | [0.000] | [0.000] | [0.000] | [0.000] |
| Midwest | -0.040 | -0.043 | -0.019 | -0.034 |
|  | [0.017]** | [0.014]*** | [0.016] | [0.014]** |
| South | -0.017 | -0.040 | -0.025 | -0.021 |
|  | [0.015] | [0.012]*** | [0.014]* | [0.012]* |
| West | -0.037 | -0.043 | -0.042 | -0.034 |
|  | [0.016]** | [0.013]*** | [0.016]*** | [0.013]** |
| Constant | 0.522 | 0.540 | 0.602 | 0.547 |
|  | [0.039]*** | [0.029]*** | [0.034]*** | [0.030]*** |
| Observations | 2529 | 2527 | 2528 | 2419 |
| R-squared | 0.123 | 0.149 | 0.131 | 0.086 |
| Mean of DV | 0.762 | 0.673 | 0.723 | 0.604 |
| Std. Dev. of DV | 0.289 | 0.231 | 0.280 | 0.223 |
| Robust standard errors in brackets |  |  |  |  |
| * significant at 10%; ** significant at 5%; *** significant at 1% |  |  |  |  |

OLS regression analysis with Huber-White standard errors for estimates displayed in Figure 1.





Distribution of scale outcomes for Experiment 1
