# Supplementary material for: Testing persuasive messaging to encourage COVID-19 risk reduction
Source: PLoS One. 2022 Mar 23;17(3):e0264782. doi: 10.1371/journal.pone.0264782 (PMC8942219; doi:10.1371/journal.pone.0264782)
Supplement: S3 Appendix — (DOCX) [file pone.0264782.s003.docx]

S3 Appendix: Subgroup Analyses for Experiment 1





Experiment 1. Covariate-adjusted treatment effect estimates for primary outcomes by age. Comparison to baseline control with 95% confidence intervals are shown.





Experiment 1. Covariate-adjusted treatment effect estimates for primary outcomes by gender. Comparison to baseline control with 95% confidence intervals are shown.




Experiment 1. Covariate-adjusted treatment effect estimates for primary outcomes by partisanship. Comparison to baseline control with 95% confidence intervals are shown.


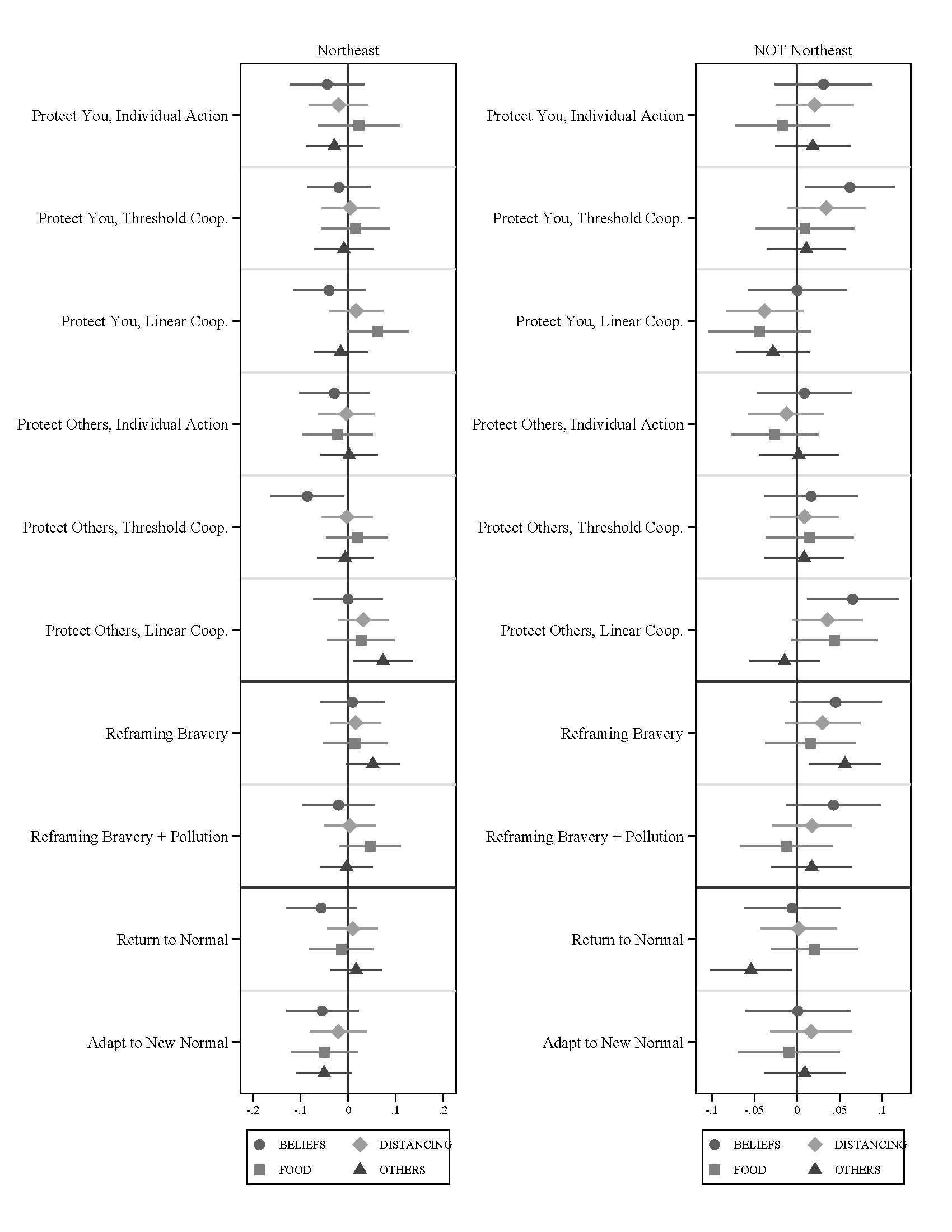


Experiment 1. Covariate-adjusted treatment effect estimates for primary outcomes split by northeast U.S. that was heavily affected by COVID-19 or not northeast. Comparison to baseline control with 95% confidence intervals are shown.





Experiment 1. Covariate-adjusted treatment effect estimates for primary outcomes by level of endorsement of liberty values. Comparison to baseline control with 95% confidence intervals are shown.
