# Supplementary material for: Testing persuasive messaging to encourage COVID-19 risk reduction
Source: PLoS One. 2022 Mar 23;17(3):e0264782. doi: 10.1371/journal.pone.0264782 (PMC8942219; doi:10.1371/journal.pone.0264782)
Supplement: S4 Appendix — (DOCX) [file pone.0264782.s004.docx]

S4 Appendix: Experiment 2 Outcome Measures

For all outcome measures composed of multiple items, we used mean imputation for missing items if the missing items constitute 25% or fewer of the measures used to construct the scale.

In the below lists where multiple items are combined to form a scale, we list the prompt used for each item first.

BELIEFS about the efficacy and importance of social distancing scale, composed of the average of the following 3 items. (Cronbach’s Alpha = 0.89)

To what extent do you agree with the following statements?

Scored 0 (Strongly disagree), .25 (Somewhat disagree), .5 (Neither agree nor disagree), .75 (Somewhat agree), and 1 (Strongly agree).

1. Practicing social distancing is important so that I do not get sick

2. Practicing social distancing is important so that I do not risk infecting others or pose a burden on the health system

3. I would feel guilty if I did not practice social distancing

DISTANCING scale is composed of the average of the following 16 items. (Cronbach’s Alpha = 0.88)

To what extent do you agree with the following statements?

Scored 0 (Strongly disagree), .25 (Somewhat disagree), .5 (Neither agree nor disagree), .75 (Somewhat agree), and 1 (Strongly agree).

1. I am likely to practice social distancing in the future

How likely are you to do each of the following things in the next two weeks?

Scored 0 (Extremely unlikely), .25 (Somewhat unlikely), .5 (Neither likely nor unlikely), .75 (Somewhat likely), and 1 (Extremely likely). For Reverse Coded items, scale is flipped.

2. Attend indoor group religious services, for example at a church, synagogue, or a mosque (Reverse Coded)

3. Go to a library (Reverse Coded)

4. Go to a doctor for an elective procedure (Reverse Coded)

5. Go inside at another family member member’s house (Reverse Coded)

6. Go inside at a friend’s house (Reverse Coded)

7. Attend a campaign rally or political protest (Reverse Coded)

8. Use public transportation, taxi, or ride share service (Reverse Coded)

9. Travel on an airplane (Reverse Coded)

10. Stay at home if you have had close unprotected contact in the last 14 days with someone who has COVID-19

11. Work outside the home (Reverse Coded)

Suppose you were diagnosed with COVID-19. How likely would you be to...

Scored 0 (Extremely unlikely), .25 (Somewhat unlikely), .5 (Neither likely nor unlikely), .75 (Somewhat likely), and 1 (Extremely likely). For Reverse Coded items, scale is flipped.

12. Alert public health authorities

In the next month, how likely would you be to attend...

Scored 0 (Extremely unlikely), .25 (Somewhat unlikely), .5 (Neither likely nor unlikely), .75 (Somewhat likely), and 1 (Extremely likely). For Reverse Coded items, scale is flipped.

13. A 5-10 person gathering **outside** at a friend's house (Reverse Coded)

14. A 5-10 person gathering **inside** at a friend's house (Reverse Coded)

15. A gathering of over 25 people **outside** at a friend's house (Reverse Coded)

16. A gathering of over 25 people **inside** at a friend house (Reverse Coded)

FOOD behaviors scale, composed of the average of the following 4 items. (Cronbach’s Alpha = 0.78)

How likely are you to do each of the following things in the next two weeks?

Scored 0 (Extremely unlikely), .25 (Somewhat unlikely), .5 (Neither likely nor unlikely), .75 (Somewhat likely), and 1 (Extremely likely). For Reverse Coded items, scale is flipped.

1. Go to a coffee shop (Reverse Coded)

2. **Eat outside** at a restaurant (Reverse Coded)

3. **Eat inside** at a restaurant (Reverse Coded)

4. Go to a bar (Reverse Coded)

Evaluations and actions toward OTHERS based on their social distancing behavior scale, composed of the average of the following 8 items. (Cronbach’s Alpha = 0.90)

How likely are you to do each of the following things:

Scored 0 (Extremely unlikely), .25 (Somewhat unlikely), .5 (Neither likely nor unlikely), .75 (Somewhat likely), and 1 (Extremely likely)

1. Report a local business that is violating rules for protecting public health, like limiting crowd sizes and maintaining appropriate social distancing

2. Persuade a friend or relative to practice social distancing if they were not already doing so

Now, we would like you to think about a friend or relative who hasn’t been practicing social distancing. What would you think about this person? Are they…

Scored 0 (Not at all), .25 (Slightly), .5 (Somewhat), .75 (Mostly), and 1 (Very). For Reverse Coded items, scale is flipped.

3. Trustworthy (Reverse coded)

4. Selfish

5. Likeable (Reverse coded)

6. Competent (Reverse coded)

7. Intelligent (Reverse coded)

Suppose you were diagnosed with COVID-19. How likely would you be to...

Scored 0 (Extremely unlikely), .25 (Somewhat unlikely), .5 (Neither likely nor unlikely), .75 (Somewhat likely), and 1 (Extremely likely).

8. If asked, share with public health authorities the names of people you might have exposed to COVID-19

MASK wearing scale, composed of the average of the following 7 items. (Cronbach’s Alpha = 0.87)

Suppose you had to do each of the following things in the next two weeks. How likely would you be to wear a mask for each activity?

Scored 0 (Extremely unlikely), .25 (Somewhat unlikely), .5 (Neither likely nor unlikely), .75 (Somewhat likely), and 1 (Extremely likely).

1. Work outside the home

2. Use public transportation, a taxi, or a ride share service

3. Go on a walk in your neighborhood

4. Shop inside a store

5. Visit inside a friend's house

6. Visit a park

The last scale component is calculated from two items asked using this prompt:

How likely are you to do each of the following things in the next two weeks?

Scored 0 (Extremely unlikely), .25 (Somewhat unlikely), .5 (Neither likely nor unlikely), .75 (Somewhat likely), and 1 (Extremely likely). For Reverse Coded items, scale is flipped.

7. The difference between these two items: Go to a store that **requires** mask wearing minus Go to a store that **prohibits** mask wearing, adding 1 to the difference and dividing by 2 so the difference runs from 0 to 1.
