# Supplementary material for: Testing persuasive messaging to encourage COVID-19 risk reduction
Source: PLoS One. 2022 Mar 23;17(3):e0264782. doi: 10.1371/journal.pone.0264782 (PMC8942219; doi:10.1371/journal.pone.0264782)
Supplement: S5 Appendix — (DOCX) [file pone.0264782.s005.docx]

S5 Appendix: Regression Results for Figure 2 and Figure 3 and Distribution of Outcomes for Experiment 2

|  | (1) | (2) | (3) | (4) | (5) |
| --- | --- | --- | --- | --- | --- |
|  | BELIEFS | DISTANCING | FOOD | OTHERS | MASKS |
| Baseline Info. | 0.023 | 0.013 | 0.011 | 0.016 | 0.011 |
|  | [0.009]** | [0.006]** | [0.009] | [0.008]* | [0.008] |
| Other Regarding, Linear Coop. | 0.029 | 0.020 | 0.007 | 0.023 | 0.018 |
|  | [0.009]*** | [0.006]*** | [0.009] | [0.008]*** | [0.008]** |
| Reframing Bravery | 0.032 | 0.019 | 0.007 | 0.030 | 0.016 |
|  | [0.009]*** | [0.006]*** | [0.008] | [0.008]*** | [0.008]* |
| Constant | 0.752 | 0.531 | 0.525 | 0.533 | 0.659 |
|  | [0.026]*** | [0.018]*** | [0.025]*** | [0.023]*** | [0.022]*** |
| Observations | 5295 | 6066 | 6067 | 6058 | 6052 |
| R-squared | 0.170 | 0.225 | 0.160 | 0.262 | 0.228 |
| Control Group Mean of DV | 0.813 | 0.711 | 0.718 | 0.574 | 0.687 |
| ORLC v Baseline est | 0.005 | 0.007 | -0.004 | 0.008 | 0.007 |
| ORLC v Baseline p | 0.274 | 0.136 | 0.342 | 0.182 | 0.192 |
| Not Bravery v Baseline est | 0.009 | 0.007 | -0.003 | 0.014 | 0.004 |
| Not Bravery v Baseline p | 0.159 | 0.141 | 0.350 | 0.047 | 0.293 |
| Average ORLC/Not Bravery v Baseline est | 0.007 | 0.007 | -0.003 | 0.011 | 0.006 |
| Average ORLC/Not Bravery v Baseline p | 0.181 | 0.106 | 0.324 | 0.069 | 0.208 |
| Covariates omitted. OLS Coefficients with robust standard errors in brackets. | |  |  |  |  |
| * significant at 10%; ** significant at 5%; *** significant at 1% |  |  |  |  |  |

OLS regression analysis with Huber-White standard errors for estimates displayed in Figure 2.

|  | (1) | (2) | (3) | (4) | (5) | (6) | (7) |
| --- | --- | --- | --- | --- | --- | --- | --- |
|  | Agree: I am likely to practice Soc. Dist. (Scale 0-1) | How likely: Stay at home if exposed to COVID-19 (Scale 0-1) | How likely: Alert authorities if get COVID-19 (Scale 0-1) | How likely: If asked, cooperate with public health authorities to trace exposure | RC How likely: Attend indoor group religious services (Scale 0-1) | RC How likely: Go INSIDE at another family member's house (Scale 0-1) | RC How likely: Go INSIDE at a friend's house (Scale 0-1) |
| Baseline Info. | 0.020 | 0.004 | 0.010 | 0.009 | 0.018 | 0.006 | 0.019 |
|  | [0.010]** | [0.010] | [0.012] | [0.011] | [0.012] | [0.013] | [0.013] |
| Other Regarding, Linear Coop. | 0.029 | 0.006 | 0.028 | 0.027 | 0.015 | 0.031 | 0.040 |
|  | [0.010]*** | [0.010] | [0.012]** | [0.011]** | [0.012] | [0.013]** | [0.013]*** |
| Reframing Bravery | 0.028 | 0.020 | 0.029 | 0.017 | 0.037 | 0.015 | 0.035 |
|  | [0.009]*** | [0.010]** | [0.011]** | [0.011] | [0.012]*** | [0.013] | [0.013]*** |
| Constant | 0.728 | 0.653 | 0.537 | 0.609 | 0.540 | 0.358 | 0.356 |
|  | [0.027]*** | [0.029]*** | [0.032]*** | [0.030]*** | [0.033]*** | [0.035]*** | [0.034]*** |
| Observations | 5766 | 6056 | 6062 | 6063 | 6060 | 6064 | 6060 |
| R-squared | 0.144 | 0.111 | 0.117 | 0.142 | 0.170 | 0.101 | 0.117 |
| Control Group Mean of DV | 0.794 | 0.814 | 0.696 | 0.750 | 0.733 | 0.454 | 0.527 |
| ORLC v Baseline est | 0.009 | 0.002 | 0.018 | 0.018 | -0.003 | 0.025 | 0.021 |
| ORLC v Baseline p | 0.187 | 0.441 | 0.061 | 0.046 | 0.412 | 0.029 | 0.049 |
| Not Bravery v Baseline est | 0.008 | 0.015 | 0.019 | 0.008 | 0.019 | 0.010 | 0.016 |
| Not Bravery v Baseline p | 0.200 | 0.063 | 0.050 | 0.224 | 0.054 | 0.229 | 0.100 |
| Average ORLC/Not Bravery v Baseline est | 0.008 | 0.008 | 0.018 | 0.013 | 0.008 | 0.017 | 0.019 |
| Average ORLC/Not Bravery v Baseline p | 0.160 | 0.170 | 0.033 | 0.080 | 0.213 | 0.064 | 0.045 |
| Covariates omitted. OLS Coefficients with robust standard errors in brackets. | |  |  |  |  |  |  |
| * significant at 10%; ** significant at 5%; *** significant at 1% |  |  |  |  |  |  |  |

OLS regression analysis with Huber-White standard errors for estimates of specific behaviors shown in Figure 3.


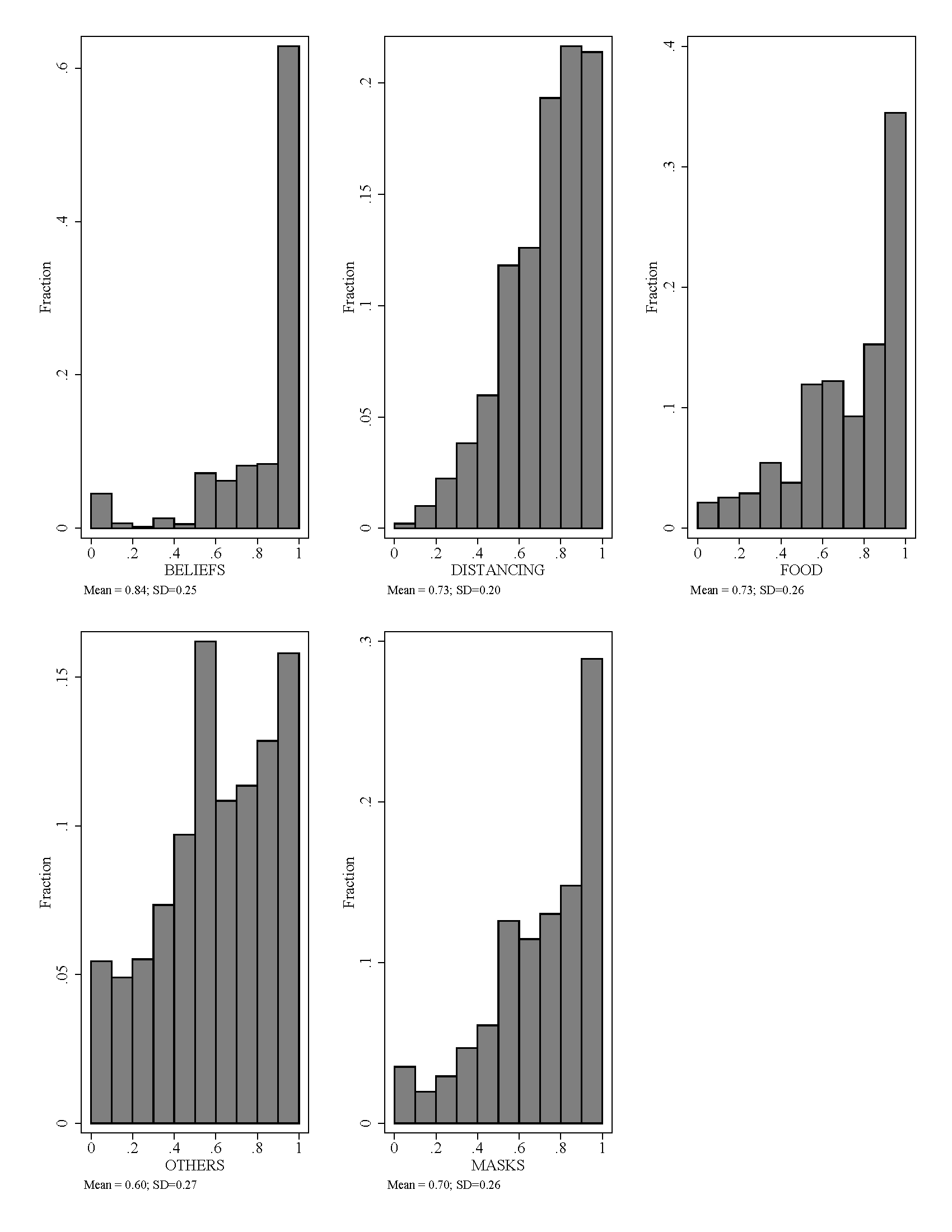


Distribution of scale outcomes for Experiment 2
