# Supplementary material for: Testing persuasive messaging to encourage COVID-19 risk reduction
Source: PLoS One. 2022 Mar 23;17(3):e0264782. doi: 10.1371/journal.pone.0264782 (PMC8942219; doi:10.1371/journal.pone.0264782)
Supplement: S6 Appendix — (DOCX) [file pone.0264782.s006.docx]

S6 Appendix: Liberty Endorsement Subgroup Analysis for Experiment 2


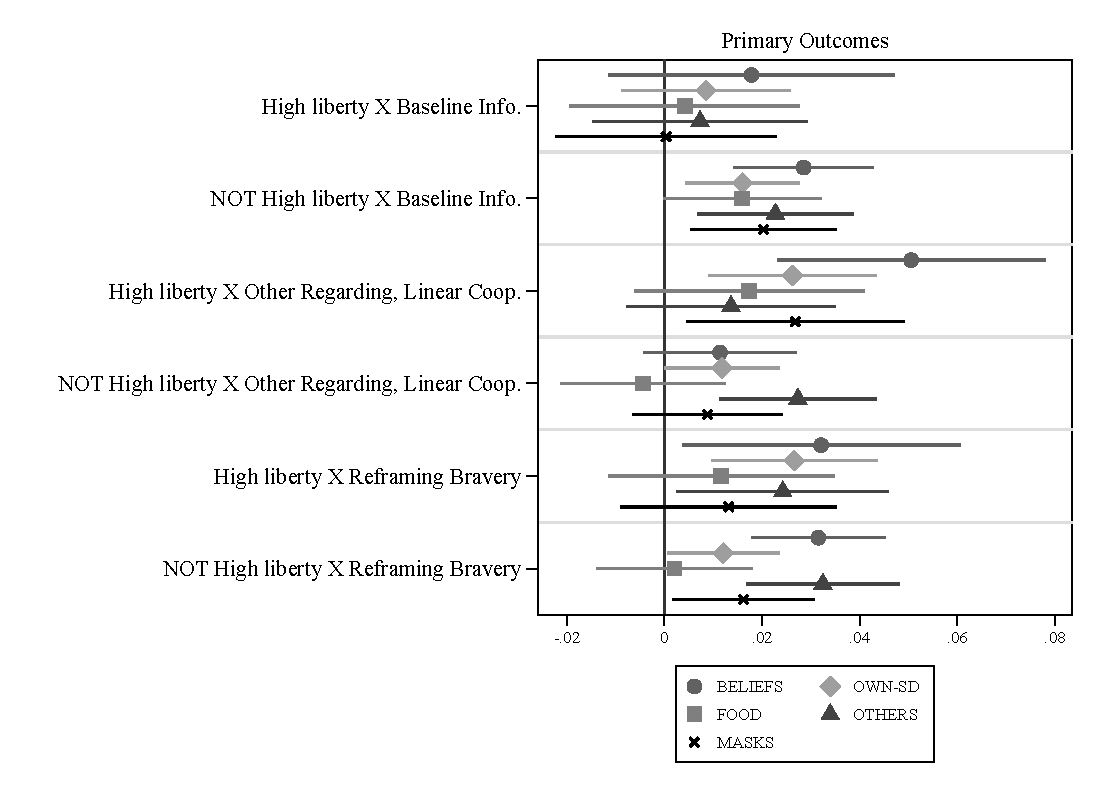


Covariate adjusted treatment effects by liberty endorsement. OLS regression estimates with 95% confidence intervals.
